# A Remote Sensor System Based on TDLAS Technique for Ammonia Leakage Monitoring

**DOI:** 10.3390/s21072448

**Published:** 2021-04-02

**Authors:** Hongbin Lu, Chuantao Zheng, Lei Zhang, Zhiwei Liu, Fang Song, Xiuying Li, Yu Zhang, Yiding Wang

**Affiliations:** State Key Laboratory of Integrated Optoelectronics, College of Electronic Science and Engineering, Jilin University, 2699 Qianjin Street, Changchun 130012, China; luhb18@mails.jlu.edu.cn (H.L.); zhangl19@mails.jlu.edu.cn (L.Z.); zhiwei18@mails.jlu.edu.cn (Z.L.); songfang16@mails.jlu.edu.cn (F.S.); yuzhang@jlu.edu.cn (Y.Z.); ydwang@jlu.edu.cn (Y.W.)

**Keywords:** tunable diode laser absorption spectroscopy, wavelength modulation spectroscopy, *2f/1f* signal processing technique, wavelength-locked, remote sensor

## Abstract

The development of an efficient, portable, real-time, and high-precision ammonia (NH_3_) remote sensor system is of great significance for environmental protection and citizens’ health. We developed a NH_3_ remote sensor system based on tunable diode laser absorption spectroscopy (TDLAS) technique to measure the NH_3_ leakage. In order to eliminate the interference of water vapor on NH_3_ detection, the wavelength-locked wavelength modulation spectroscopy technique was adopted to stabilize the output wavelength of the laser at 6612.7 cm^−1^, which significantly increased the sampling frequency of the sensor system. To solve the problem in that the light intensity received by the detector keeps changing, the *2f/1f* signal processing technique was adopted. The practical application results proved that the *2f/1f* signal processing technique had a satisfactory suppression effect on the signal fluctuation caused by distance changing. Using Allan deviation analysis, we determined the stability and limit of detection (LoD). The system could reach a LoD of 16.6 ppm·m at an average time of 2.8 s, and a LoD of 0.5 ppm·m at an optimum averaging time of 778.4 s. Finally, the measurement result of simulated ammonia leakage verified that the ammonia remote sensor system could meet the need for ammonia leakage detection in the industrial production process.

## 1. Introduction

With the continuous development of industrialization, the application of ammonia (NH_3_) in industry has become more and more common, especially in the petrochemical industry, cold chain logistics, and fertilizer production. At the same time, traditional animal husbandry and agriculture are also important sources of ammonia emissions. SO_2_ and NO_2_ in the atmosphere react with NH_3_ under the action of water vapor to produce ammonium sulfate and ammonium nitrate particles. These particles are suspended in the atmosphere to form the PM2.5 aerosol, which is an important reason for the formation of haze [1,2]. As a major chemical and agricultural production country around the world, China emits about 13.1 million tons of NH_3_ into the atmosphere each year, making it the largest NH_3_ emitter in the world [3]. Therefore, the development of an efficient, convenient, real-time, and high-precision NH_3_ remote sensor system is of great significance to improve the level of environmental protection and citizens’ health.

As a key subject, researchers from various countries have proposed many methods to measure gas concentration [4,5,6,7,8,9,10,11,12]. As for the remote sensing of ammonia, Force et al. achieved the sensitivity of 5 ppb for atmospheric NH_3_ measurement by use of a CO_2_ differential absorption lidar system with the wavelength of 10.716 μm and 10.693 μm [13]; Theriault et al. designed a compact atmospheric sounding interferometer (CATSI) which could achieve the resolution of 40 ppm·m for NH_3_ remote sensing based on differential Fourier-transform infrared (FTIR) spectroscopy [14]; Dammers et al. did a lot of work on the research of the spatial distribution of NH_3_ with mini Differential Optical Absorption Spectroscopy (mini-DOAS) [15,16,17]. Dror et al. introduced an encoding–decoding method to improve spectral resolution when detecting various chemical species with Raman spectroscopy [18]. Comparing these methods, gas detection systems based on tunable diode laser absorption spectroscopy (TDLAS) technique have the merit of high resolution. However, traditional NH_3_ detection equipment based on TDLAS is usually deployed with a gas cell inside the device. Furthermore, NH_3_ will adhere to the pipeline and the system, which affects the accuracy, and the corrosion of pipelines also affects the life of the equipment. [19]. At the same time, the equipment deployed with a gas cell makes the testers inevitably exposed to the environment of NH_3_ leakage, which is not satisfactory from the perspective of industrial production. With the development of laser and waveguide technology, infrared laser beam can propagate over long distance after collimation. This is also the foundation for infrared laser remote sensing technology to achieve application [20].

In this paper, we propose a remote sensor system for NH_3_ leakage monitoring in the industrial production process based on the TDLAS technique. This system avoided deploying the gas cell which was usually used in traditional TDLAS sensor system. This change solved the problem caused by the adsorption and corrosion of the ammonia. Combined with the *2f/1f* wavelength modulation spectroscopy (WMS) technique, this system suppressed the interference caused by the varied intensity and detection distance retained the merit of high resolution of TDLAS gas sensor system. To improve the refresh rate of digital system, we gave up the traditional “triangular wave + sine-wave” WMS scheme and adopted of “wavelength lock” scheme. This change means the system can scan the absorption line of NH_3_ much more rapidly than the traditional TDLAS gas sensor. To realize the portability, we miniaturized this system into a handheld instrument. Considering the convenience of maintenance, later performance upgrade, and the cost of the system, we developed the digital lock-in amplification and *2f/1f* signal processing program based on LabVIEW platform. We carried out a series of instrument performance characterization experiments and outdoor practical application tests, which verified the system has satisfactory performance. The NH_3_ remote sensor system has the advantages of simple structure, low cost, excellent portability, rapid response, remote no-contact measurement, and excellent anti-interference performance, which has promising application prospects.

## 2. The Detection Theory and System Structure

### 2.1. 2f/1f WMS Signal Processing Technique

The vibrational absorption spectra of the chemical bonds of gas molecules are located in the mid-infrared band (2.5–300 μm). The overtone of these absorption spectra form the near-infrared spectra (0.78–2.5 μm) of the gas molecules. Different molecules have their own fingerprint region [21,22]. When the laser beam passes through the measured gas, its light intensity attenuation follows the Lambert–Beer law, which is expressed as:

(1) $$I\left(v\right)=\eta I_{0}\left(v\right)\exp\left[-\alpha \left(v\right)L\right]$$

when α(v)L << 1 (or α(v)L < 0.1), the formula is given by:

(2) $$I\approx \eta I_{0}\left(v\right)\left(1-\alpha \left(v\right)L\right)$$

where *η* is the reflection index of the target, $I_{0}\left(v\right)$ is the intensity of laser output, I(v) is the intensity of signal received by the remote sensor system. $\alpha \left(v\right)=P\chi_{NH_{3}}S_{NH_{3}}\varphi_{NH_{3}}$ where *P* is gas pressure, $\chi_{NH_{3}}$ is NH_3_ concentration, $S_{NH_{3}}$ and $\varphi_{NH_{3}}$ are the line strength and line shape function of the absorption line, respectively [23].

According to WMS, the laser output wavelength can be modulated by modulating the injection current of the laser. The output frequency of the laser and the intensity are given by [24]:

(3) $$v\left(t\right)=\bar{v}+acos\left(\omega t\right)$$

(4) $$I_{0}\left(t\right)=\bar{I_{0}}\left(1+i_{1}cos\left(\omega t+\psi_{1}\right)\right)$$

where *a* is the modulation depth, *i*_1_ is the linear intensity modulation coefficient, and *ψ*_1_ is the intensity modulation phase shift.

Since the spectral absorption coefficient α(v) is a periodic even function of the modulation frequency *ω*, it can be expanded in a Fourier cosine series at its central frequency $\bar{v}$:

(5) $$-\alpha \left(\bar{v}+acos\left(\omega t\right)\right)L=\overset{\infty }{\underset{k=0}{\sum }}H_{k}\left(\bar{v},a\right)cos\left(k\omega t\right)$$

where the *k*_th_ Fourier coefficients are given by:

(6) $$H_{0}\left(\bar{v},a\right)=\frac{P\chi_{NH_{3}}L}{2\pi }\int_{-\pi }^{\pi }\sum S_{NH_{3}}\varphi_{NH_{3}}\left(\bar{v}+acos\left(\omega t\right)\right)d\omega t$$

(7) $$H_{k}\left(\bar{v},a\right)=\frac{P\chi_{NH_{3}}L}{2\pi }\int_{-\pi }^{\pi }\sum S_{NH_{3}}\varphi_{NH_{3}}\left(\bar{v}+acos\left(\omega t\right)\right)cos\left(k\omega t\right)d\omega t$$

From Equations (6) and (7), it can be seen that the amplitude of each harmonic component is proportional to the integral concentration of the measured gas.

During the process of remote sensing, the reflective target is not fixed, and its reflectivity cannot be accurately known either. At the same time, as the remote sensing distance increases, the strength of the signal received by the receiver gradually weakens. In this case, we got the first harmonic and second harmonic by using a lock-in amplifier and normalized the second harmonic with the first harmonic. This is the so-called *2f/1f* signal processing technique. The influence of changing signal intensity on the detection results can be effectively eliminated in this way. The amplitude of the second harmonic extracted and amplified by the lock-in amplifier can be expressed as [23,24]:

(8) $$S_{2f}={\left(X_{2f}{}^{2}+Y_{2f}{}^{2}\right)}^{1/2}=\frac{\eta G\bar{I_{0}}}{2}{\left\{{\left[H_{2}+\frac{i_{1}}{2}\left(H_{1}+H_{3}\right)cos\psi_{1}\right]}^{2}+{\left[\frac{i_{1}}{2}\left(H_{1}-H_{3}\right)sin\psi_{1}\right]}^{2}\right\}}^{1/2}$$

where *G* is the optical-electrical gain of the detection system. Since the measured gas is under standard atmospheric pressure, the broadening of the absorption spectrum shows a Lorentzian profile. The Fourier series of the Lorentz profile is an even harmonic function which means the amplitude of the odd harmonic component is zero [25,26]. So, Equation (8) will be simplified to:

(9) $$S_{2f}=\frac{\eta G\bar{I_{0}}}{2}H_{2}$$

In the same way, the equation of the first harmonic is given by:

(10) $$S_{1f}={\left(X_{1f}{}^{2}+Y_{1f}{}^{2}\right)}^{1/2}=\frac{\eta G\bar{I_{0}}}{2}{\left\{{\left[H_{1}+i_{1}\left(1+H_{0}+\frac{H_{2}}{2}\right)cos\psi_{1}\right]}^{2}+{\left[i_{1}\left(1+H_{0}-\frac{H_{2}}{2}\right)sin\psi_{1}\right]}^{2}\right\}}^{1/2}$$

where *H*_1_ = 0, and for low concentration gas, *H*_0_, *H*_2_ << 1, thus:

(11) $$S_{1f}=\frac{\eta G\bar{I_{0}}}{2}i_{1}$$

(12) $$\frac{S_{2f}}{S_{1f}}=\frac{H_{2}}{i_{1}}$$

It can be seen from Equation (12) that using 1*f* signal to normalize 2*f* signal can eliminate the influence of various interference on the remote sensing results, such as the change of target reflectivity, the change of detection distance, the temperature drift of the photoelectric diode, and the change of the output laser light intensity, which greatly improves the anti-interference performance.

### 2.2. NH_3_ Absorption Line Selection

**Figure 1:**
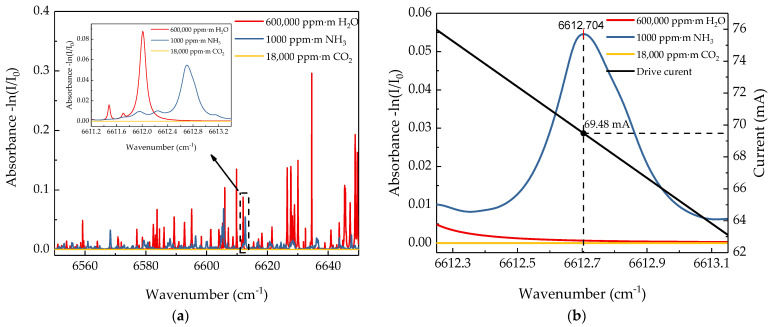
(**a**) Absorption spectra of NH_3_, H_2_O, CO_2_ from 6550 cm^−1^ to 6650 cm^−1^. (**b**) Absorption spectra of NH_3_ (1000 ppm·m, blue line), H_2_O (1%, red line), and CO_2_ (0.03%, yellow line) at an optical path of 60 m based on HITRAN database and the plot of drive current versus wavenumber of the distributed feedback (DFB) laser (black line), the value of modulation current at the central wavenumber 6612.7 cm^−1^ is 69.48 mA.

According to the HITRAN database, H_2_O and CO_2_ in the atmosphere are the main interferences to the NH_3_ absorption line. For the remote sensor system, although the absorption intensity of NH_3_ molecules at 1512 nm nearby is several orders of magnitude higher than that of H_2_O and CO_2_ molecules [27], affected by the detection distance, the integral concentration of H_2_O molecules through the entire remote sensing range is much higher than that of NH_3_ molecules. Figure 1a shows the absorption spectra of NH_3_, H_2_O, and CO_2_ from 6550 cm^−1^ to 6650 cm^−1^ based on the HITRAN database. It is clearly shown that the absorption line at 6612.7 cm^−1^ is the strongest line of NH_3_ around the range and has no overlap with the absorption lines of H_2_O and CO_2_. Furthermore, it is a single-peak absorption line which satisfies the requirement of *2f/1f* WMS signal processing theory. So, the characteristic absorption line at 1512.2 nm (wavenumber = 6612.7 cm^−1^) is selected as the wavelength of the remote sensing laser. Figure 1b shows the absorption intensity of 1000 ppm·m NH_3_, 600,000 ppm·m H_2_O and 18,000 ppm·m CO_2_ which are integral concentrations under the typical remote sensing distance of 30 m based on the HITRAN database. According to the data in Figure 1b, the absorption intensity of H_2_O and CO_2_ is much lower than that of NH_3_. Thus, the absorption intensity of H_2_O has no effect on the NH_3_ sensing results. Considering the absorption line of H_2_O at 6612 cm^−1^ interfering to the characteristic absorption line of NH_3_ at 6612.7 cm^−1^, the traditional “triangular wave + sine-wave” WMS scheme is abandoned and the “wavelength lock” scheme is adopted instead. This scheme is to fix the static output wavelength of a distributed feedback (DFB) laser at the center frequency and just modulate the output wavelength with a sinewave signal, which not only increases the sampling frequency, but also eliminates the interference of the H_2_O absorption line on the detection results.

### 2.3. Output Characterization of DFB Laser and Wavelength-Locked Technique

**Figure 2:**
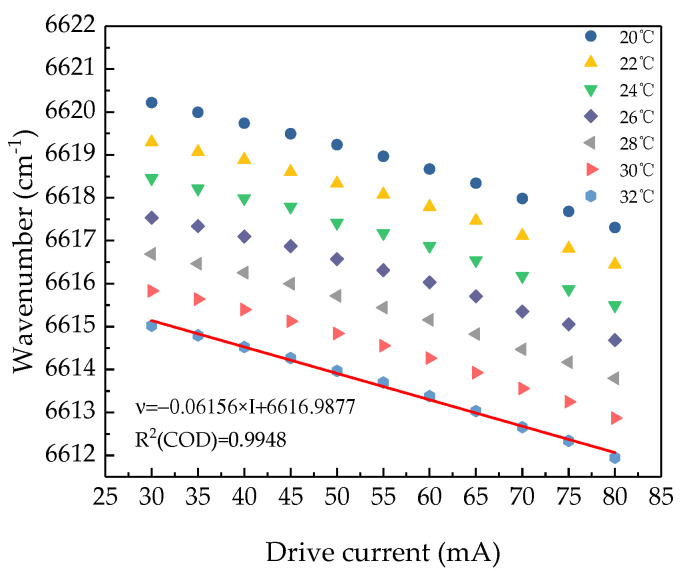
Output wavenumber of the DFB laser versus drive current at 20 °C, 22 °C, 24 °C, 26 °C, 28 °C, 30 °C, and 32 °C. The red line is the linear fitting line of the selected temperature (32 °C). The slope of the fitting line is 0.06156, which is the reciprocal of the slope of the black line shown in Figure 1b.

After the absorption line was selected, we chose a distributed feedback (DFB) laser at 1512.25 nm (Model: BF14, Sichuan Tengzhong light technology co., LTD, Mianyang, China) as the remote sensing laser source. A Fourier-transform infrared spectrometer (FTIR Spectrometer) was used to measure the characteristic parameters of the laser at different temperature and drive currents, the result is shown in Figure 2. The output wavelength of the laser is 1512.2 nm (6612.7 cm^−1^) at 32 °C when the drive current is around 70 mA. After linear fitting at this temperature, the current modulation coefficient of the DFB laser is obtained as 0.0141 nm/mA. The black line in Figure 1b shows the change of modulation current within the range of wavelength modulation according to the test result of the DFB laser at 32 °C. It can be seen from the figure that when the drive current is 69.48 mA, the output wavelength is located at the center of the NH_3_ characteristic absorption line at 6612.7 cm^−1^. The output power of the laser is 11.28 mW at this point. Due to the interference of the H_2_O absorption line at 6612 cm^−1^, the modulation depth of the output wavelength should be less than 0.5 cm^−1^. Table 1 shows the characteristics of the selected laser. It should be noted that the characteristics are different between different laser devices and should be adjusted independently for measurement.

### 2.4. Design of Remote Sensor System Structure

**Figure 3:**
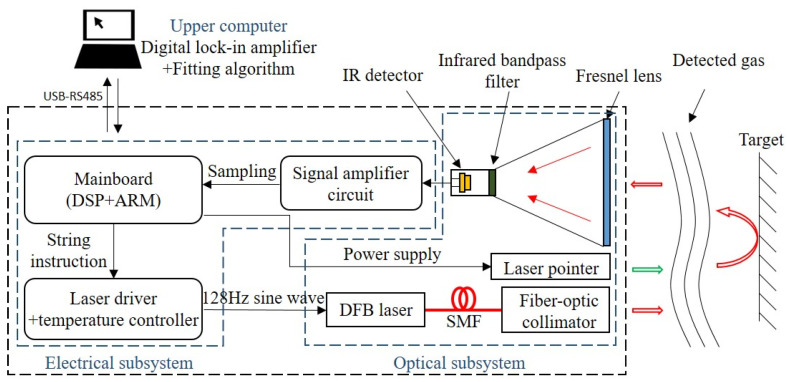
Schematic of the ammonia remote sensor system. This system consists of three parts: an optical subsystem, an electrical subsystem, and an upper computer.

Figure 3 shows the system structure of the remote sensor system. The system mainly includes three parts: the optical subsystem, the electrical subsystem, and the upper computer. The electrical subsystem is mainly composed of three parts: the main control module, the laser drive/temperature control module, and the signal amplifier module. The main control module is a self-made main control board based on DSP + ARM architecture processor. The functions of the main control board include the following: communicating with the upper computer through the RS-485 protocol with a baud rate of 115,200; sending string instructions to the laser drive module to control it to generate a modulated current; sampling and quantizing the received signal by ADC on the board, the sampling frequency of which is 4 kHz; and supplying power to the system. The laser drive receives instructions from the main control module, and the DDS chip generates a sinusoidal drive current with a frequency of 128 Hz. The temperature control circuit keeps the voltage of the thermistor packaged in the DFB laser at 1.48 V to stabilize the center wavelength of the laser at 1512.2 nm. Because each laser is slightly different, the specific voltage and current values should be adjusted corresponding to different lasers.

The optical subsystem is mainly composed of a laser, an optical fiber collimator (CFC11A-C, Thorlabs), a laser pointer, and a signal-receiving module. The DFB laser has a center wavelength of 1512 nm, which is connected to a collimator through a single-mode fiber (SMF) to generate a collimated laser beam. The collimator can adjust the emission direction of the laser and is fixed by reserved mounting seat to keep mechanical stability. This makes the optical path unchangeable when it encounters collision and vibration. Single-mode fiber has several merits in this application scenario; for example, low attenuation (about 0.2 db/km) keeps the energy after the transmission; the low dispersion coefficient keeps the wave shape after the transmission; the narrow line width of the DFB laser is suitable to transfer in SMF [20,28]. The output laser beam should be adjusted to make it parallel to the beam of laser pointer (green light) during system debugging to make sure that the remote system can accurately align the measured gas during operation. The signal receiving module is composed of a Fresnel lens, an infrared filter, an infrared detector, and an amplifying circuit. The Fresnel lens (*Φ* = 150 mm, *f* = 140 mm, profile pitch = 0.3 mm) was used to receive the reflected light signal. To ensure the strength of the received signal, the profile pitch needs to be as narrow as possible with a fixed diameter of lens; furthermore, the focal length of the Fresnel lens needs to be as long as possible if it is within the acceptable range for the remote system. A longer focal length lens facilitates to adjust the optical path of the receiving module. The infrared detector is an InGaAs infrared photodiode (PGA10, Thorlabs), which is used to convert the received light signal into an electrical signal. Since the signal receiver receives all the optical signals with various wavelengths from the external environment, a band-pass infrared filter (FB1510-12, Thorlabs) is set up to filter out all the unexpected signal except the target wavelength. This reduces the influence of background noise on the telemetry results and improves the detection accuracy of the system.

The function of the upper computer is to process the telemetry data sent by the telemeter and display the result. A data processing platform developed based on LabVIEW runs on the upper computer to receive the remote sensing data. The platform is equipped with a digital lock-in algorithm, which can extract the first and second harmonics from the remote sensing signal. The second harmonic is normalized by the first harmonic and the calculation results are statistically averaged within a frame of data domain (4096 data points). At the same time, the data processing software can make a fitting on the calculation results. The fitting results are displayed through the front end. The advantages of using the upper computer to run the data processing software allow to avoid integrating the lock-in amplifier module and the display module into the system, which can greatly reduce the complexity of the system, and drops the R&D cost and production cost of the system. Furthermore, the data processing software can run on any computer, which is convenient for the post debugging, modification, and function upgrade.

The remote sensor system we developed is highly integrated and cost-acceptable, which means it is more promising for improving productivity in commercial applications. All of the hardware was designed based on specific needs, including deleting an unnecessary module which miniaturized this system into a handheld instrument.

## 3. Results and Discussion

### 3.1. Modulation Depth Optimization

**Figure 4:**
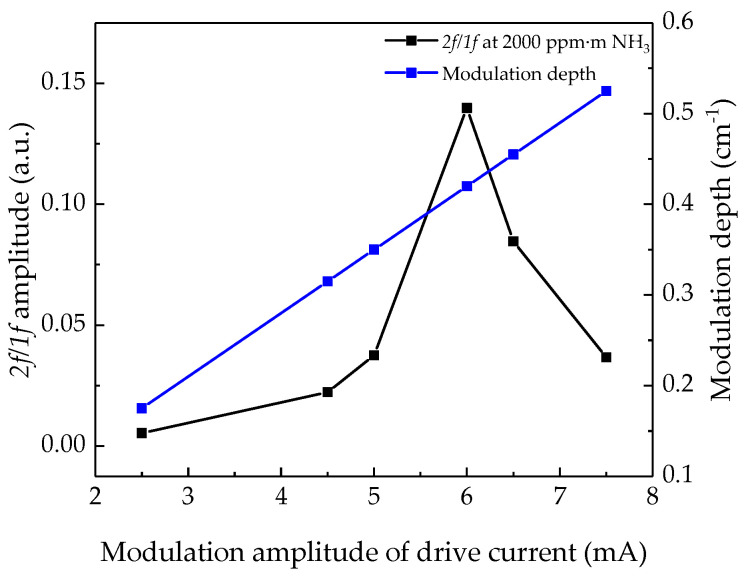
Modulation depth optimization performed at 2000 ppm∙m NH_3_, the optimal modulation depth is 0.4199 cm^−1^ with a modulation amplitude of drive current at 6 mA.

Typically, to obtain the maximum amplitude of the harmonic signal, the modulation depth should be 1.1 times greater than the full width at half maximum (FWHM) of the selected absorption line [29]. The method in the experiment is to adjust the resistor controlling the modulation current amplitude on the laser drive board to convert the modulation current amplitude into the corresponding modulation wavelength amplitude until finding the maximum of *2f/1f* signal calculated by the upper computer when measuring the gas sampling bag with the same integral concentration. The corresponding modulation wavelength amplitude of the resistor is the best modulation depth. In this experiment, a gas sampling bag with an integral concentration of 2000 ppm·m was selected to optimize the modulation depth. During the experiment, the modulation amplitude of the drive current was selected to be 2.5–7.5 mA, and the measured *2f/1f* signal amplitude is shown in Figure 4. When the amplitude of the drive current is 6 mA (modulation depth is 0.4199 cm^−1^), the *2f/1f* signal amplitude reaches the maximum. The modulation depth is 1.52 times greater than the FWHM of the NH_3_ absorption line.

### 3.2. Calibration of the NH_3_ Remote Sensor System

**Figure 5:**
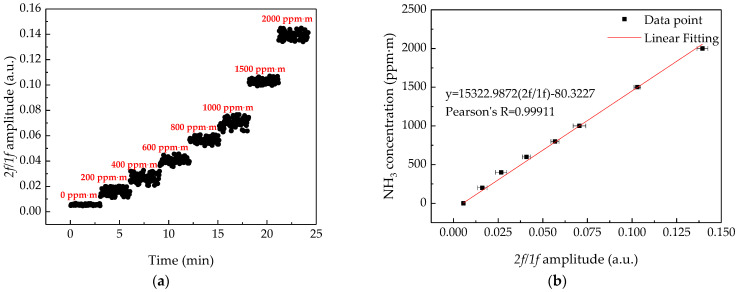
(**a**) Calibration of system by using a gas sampling bag with integral concentration from 0ppm·m to 2000 ppm·m, each concentration level was measured and recorded for about 3 min. (**b**) Measured data dots and linear fitting curve of the NH_3_ integral concentration versus *2f/1f* signal amplitude.

According to the theory and practical test of the remote system, it can be known that the amplitude of *2f/1f* signal is proportional to the integral concentration of the measured gas. To facilitate the operation of testers, the system needs to be calibrated for concentration measurement. In this experiment, the NH_3_ remote sensor was calibrated by a series of gas sampling bags (the material is Teflon [13]), with an integral concentration range from 0 ppm·m to 2000 ppm·m, and the target was a wall. The results obtained are shown in Figure 5a, the standard deviation of the result at 0 ppm·m is 4.85 × 10^−4^ ppm·m, and the standard deviation of the result at 200–2000 ppm·m is between 1.90 × 10^−3^ and 3.56 × 10^−3^ ppm·m. This was caused by the vibration of the air bag and the optical path of the air bag could not be controlled sufficiently uniformly during measurement. The integral concentration value was plotted as a function of the *2f/1f* signal with error band of *2f/1f* as shown in Figure 5b. The linear fitting of the data point was obtained as:

(13) C=15322.9872(2f/1f)−80.3227

The linear correlation coefficient is 0.99911, which indicates that the *2f/1f* signal has an excellent linear relationship with the integral concentration of gas. Note that the fluctuation at 0 ppm·m level is significantly weaker than others. The system was calibrated by using gas sampling bags; when measuring the signal amplitude of the integral concentration of 0 ppm·m, we filled the bag with nitrogen (N_2_). This meant it could be less affected by the external environment. For example, when measuring gas sampling bags with other integral concentrations, a tiny incident angle change of infrared laser beam could make the optical path length of the bag change, which caused larger fluctuation in the measurement results. However, the 0 ppm·m bag could not be changed in this situation.

### 3.3. The Effect of 2f/1f Signal Processing Technique on Sensing Results

**Figure 6:**
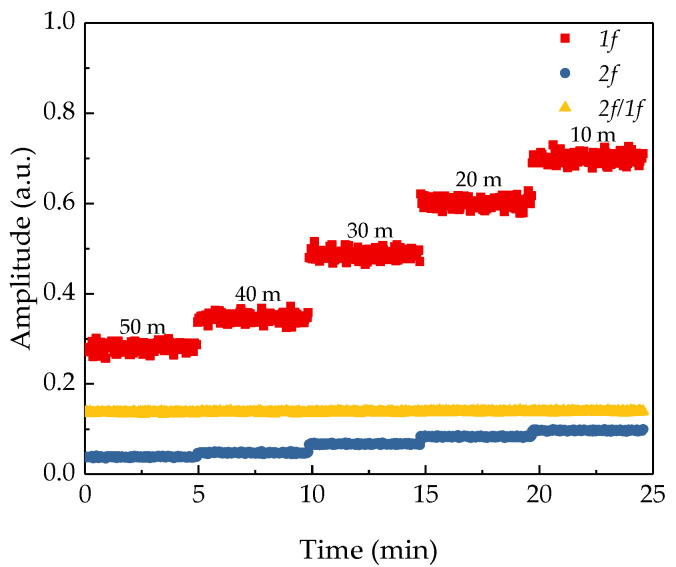
The change trend of the first harmonic, second harmonic, and the *2f/1f* signal of the absorbed signal versus the remote sensing distance.

Traditional TDLAS based sensor system usually use the second harmonic of the absorption signal to characterize the concentration of the measured gas, while the NH_3_ remote sensor system uses the first harmonic to normalize the second harmonic to reduce the interference of signal strength on remote sensing results. To verify the effect of the *2f/1f* signal processing technique on noise suppression, the amplitudes of the *1f* signal, *2f* signal and the *2f/1f* signal measured with the same gas sampling bag under different distances were recorded. The experiment was carried out in the corridor inside the building, the target was a wall, and the integral concentration of the gas sampling bag was 2000 ppm·m. During the experiment, the gas sampling bag was fixed at one end of the corridor, and the NH_3_ remote sensor system was placed on a movable cart at the other end of the corridor and the distance between the two ends was about 50 m. Pushing the cart to change the detection distance, the amplitudes of the *1f* signal, *2f* signal, and *2f/1f* signal were recorded by the NH_3_ remote sensor system at 50 m, 40 m, 30 m, 20 m, 10 m. The measurement time for each distance was 5 min; the measurement results are shown in Figure 6. It can be seen from the figure that the *1f* signal is sensitive to the change of the distance. With the increase of the distance, the amplitude of the *1f* signal keeps decreasing significantly, and the amplitude of the *2f* signal slightly changes during the entire process of the detection. However, the normalized *2f/1f* signal does not change significantly during the entire experiment, and the theoretical and experimental results are well verified.

### 3.4. System Stability and Allan Deviation

**Figure 7:**
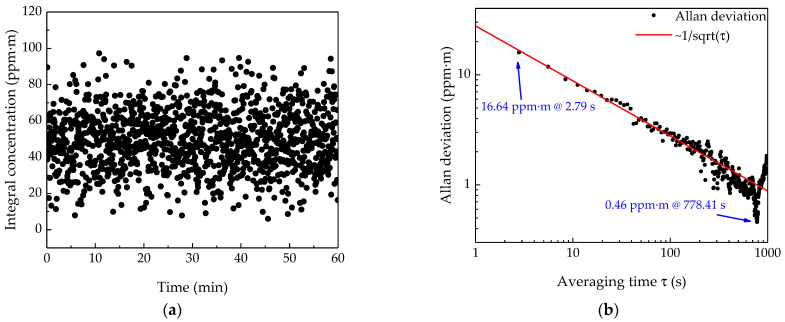
(**a**) The stability test of the remote sensor system for 1 h in the corridor of the building. (**b**) Allan deviation analysis of the sensor based on the stability test data shown in (**a**).

Allan deviation is an important tool to characterize the stability of the system. The lower limit of detection (LoD) of the system and the influence of noise on system accuracy can be obtained through Allan deviation analysis. The sampling period of the NH_3_ remote sensor is 2.79 s. The experiment measured the change of the NH_3_ concentration in the corridor of the building within 1 h. The signal amplitude detected by the system was converted to concentration via Equation (12). The results are shown in Figure 7a. As shown in the figure, the results fluctuate around 50 ppm·m. The fluctuation is caused by the background noise generated by the internal noise of the system and the change of the external environment, and the influence of the white noise component can be suppressed by averaging the results in the time domain. The Allan deviation reflects the fluctuation of the measurement result with the extension of the averaging time. Figure 7b shows the relationship between Allan deviation and the averaging time τ. When the averaging time is 2.79 s, the 1σ LoD is 16.6 ppm·m, and when the averaging time is 778.41 s, the 1σ LoD of the system reaches a minimum of 0.46 ppm·m. At the same time, when the averaging time is within 700 s, the trend of the Allan deviation decreasing is basically the same as $1/\sqrt{\tau }$, which indicates that Johnson noise is dominant in the sensing results at this time. When the averaging time reaches 700 s, increasing the averaging time cannot further improve the detection accuracy. At this time, the wavelength fluctuation noise is the main interference [30,31,32].

By further analysis of the test results we can find that the NH_3_ concentration detected in the corridor is about 50 ppm·m, which is a little higher than the normal NH_3_ concentration in air. Three potential NH_3_ sources in the corridor could cause this phenomenon: the toilet located in the corridor; the construction materials prepared for renovation of our college building; some organic synthesis experiments conducted in other laboratories in this building. Apart from these, this phenomenon is due to the system calibration. We can easily find out from Figure 5b that the linear fitting line of calibration experiment does not pass through the zero–zero point of the coordinate, which means a measuring result of zero cannot be obtained by using this fitting expression when the NH_3_ concentration is close to 0 ppm·m. In other words, this is not the best working range of the system. However, this deviation does not affect the application of the system, because this system is used to detect whether the NH_3_ concentration is beyond the national safety standard or not, which is 40 ppm for China. In this experiment, we only focus on the fluctuation of measuring result, which can characterize the stability of the system.

### 3.5. Response Time

The main factor that restricts the response time of the system is the communication rate between the sensor and the host computer. The serial port communication baud rate is 115,200 to send data to the computer. Each frame of data consists of 4096 32-bit floating point data points, including 128 modulation signal cycles. Theoretically, it takes 1.28 s to send one frame of data. Each frame of data is processed through a digital lock-in amplifier to extract the harmonic amplitude, remove the data points affected by the Fourier transform boundary conditions in the harmonic amplitude, and perform arithmetic average on the remaining 3584 data points and performs parameter fitting to generate a final telemetry result. In the practical test situation, a total of 1289 data points were obtained in 1 h of test time. Therefore, the data refresh time of the sensor is 2.79 s. This response time is based on the “wavelength lock” scheme. If we adopted the traditional “triangular wave + sine-wave” scheme of TDLAS, the drive current would be modulated by, for example, 10 Hz triangle wave and 1 kHz sine wave, which means the sensor system can only scan 20 times per second. Compared to our scheme, the wavelength-lock mode can achieve a 6 times faster refresh rate. At the same time, this scheme has a higher requirement for the temperature stability of the system to avoid wavelength drift. The limit factor for improving response time is the communication rate. If we integrate the lock-in amplifier module in the system and transfer the harmonic signal to the computer directly, this limitation can be overcome.

### 3.6. Simulated Application

**Figure 8:**
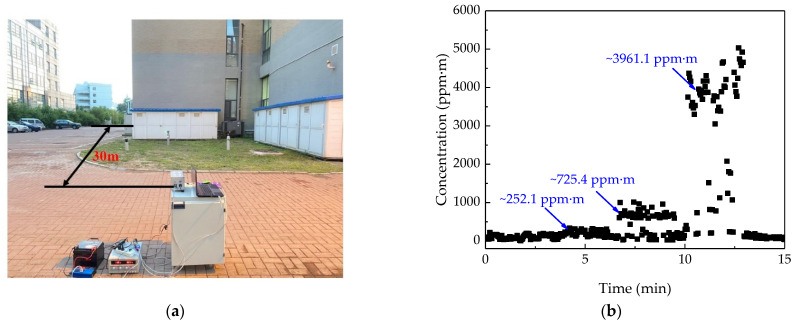
(**a**) The outdoor experiment where NH_3_ gas cylinders were set at a distance of 25 m to simulate industrial NH_3_ leakage; the concentration order from right to left is 2000 ppm, 10,000 ppm, 50,000 ppm; and the distance from target is 30 m. (**b**) The integral concentration of NH_3_ at the three leakage ports obtained by the NH_3_ remote sensor system based on the test conditions shown in (**a**).

The calibrated and debugged ammonia remote sensing system was tested outdoors for NH_3_ leakage under simulated conditions. The test environment is shown in Figure 8a. During the experiment, NH_3_ cylinders with concentrations of 50,000 ppm, 10,000 ppm, and 2000 ppm were placed from left to right at a distance of 25 m. The target was a painted steel plate at a distance of 30 m from the remote sensor system. The NH_3_ cylinder was opened during the test to simulate the NH_3_ leakage under industrial conditions, and the indicating laser of the remote sensor system was targeted at the NH_3_ leakage port, so that the system subsequently measured the NH_3_ concentration at the three leakage sources; the measurement time was about 2 min 30 s. When we finished the measurement of one leakage source, we pushed the platform to the next leakage port and targeted it vertically to keep the distance unchanged. Figure 8b shows the NH_3_ concentration of the three leakage sources measured by the system, the order of the measurement was from right to left. It can be seen from the figure that there are obvious differences in the concentration between different leakage sources. The system placed on the ground contains a lead-acid battery, an inverter, and a stabilized power supply, which simulates vehicle power supply. This experiment shows that the ammonia sensor has good practicability.

**Figure 9:**
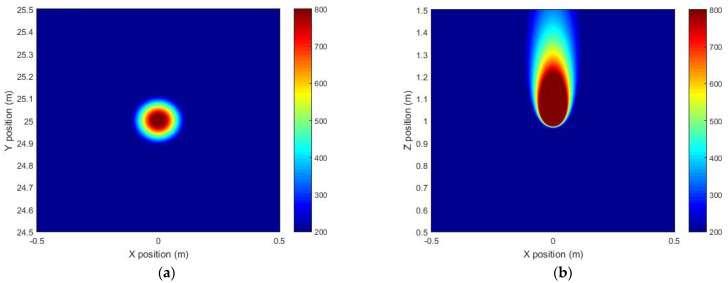
(**a**) The horizontal distribution of NH_3_ concentration 0.2 m above the leakage source. (**b**) The vertical distribution of NH_3_ concentration at the leakage source.

To analyze the precision of the system, we simulated the spatial distribution of the NH_3_ concentration under the no-wind condition based on MATLAB by use of the gas turbulence diffusion model. Figure 9a shows the horizontal distribution of NH_3_ concentration 0.2 m above the leakage source, and Figure 9b shows the vertical distribution of NH_3_ concentration at the leakage source. The concentration of leakage source is 10,000 ppm, and the flow rate is 0.05 L/s, which is close to the actual situation. The integral concentration of NH_3_ at 0.2 m above the leakage source is 995 ppm·m according to the simulated result. The tested stable value is 725.4 ppm·m in the experiment. Considering the environmental influence on the experiment, the measurement result is consistent with the theoretical diffusion model, indicating that the remote sensor system has satisfactory precision.

## 4. Conclusions

This paper introduced a NH_3_ remote sensor system based on the TDLAS technique. To solve the problems caused by the target reflectivity and light intensity change during remote sensing, the *2f/1f* signal processing technique and its mathematical principles were introduced. According to the drive characteristics of the laser, the static output is stabilized at 6612.7 cm^−1^ by controlling the temperature and the driving current. Based on the modulation depth optimization and concentration calibration, the effect of the *2f/1f* signal processing technique on the remote sensing results under practical test conditions were presented. The results showed that the *2f/1f* signal processing technique has a good inhibitory effect on the fluctuation of the remote detection results. Allan deviation analysis was used to characterize the stability of the system. It was concluded that when the averaging time was 2.79 s, the 1σ LoD of the system was 16.6 ppm·m, and when the average time was 778.41 s, the 1σ LoD of the system reached a minimum of 0.46 ppm·m. The detection limit met Chinese safety standard for indoor NH_3_ concentration of 40 ppm. The response time of the system was measured to be 2.79 s. Three NH_3_ cylinders were used to simulate the leakage source to carry out an experiment of leakage measurement under outdoor conditions, and satisfactory remote sensing results were obtained.

## Figures and Tables

**Table 1:** The typical value and test value of the selected DFB laser characteristics.

Name	Unit	Min	Typical Value	Max	Test Data
Threshold Current	mA	--	10	18	9
Output Power	mW	10	12	--	11.28
Operating Current	mA	--	70	100	69.40
Operating Voltage	V	--	1.5	2	1.48
Temperature	°C	15	25	35	32
Slope	W/A	0.05	0.1	--	0.1
Peak Wavelength	nm	1512.15	1512.25	1512.35	1512.24

## Data Availability

Not applicable.
